# Patient with toxoplasmosis and glucose-6-phosphate dehydrogenase deficiency: a case report

**DOI:** 10.4076/1757-1626-2-8826

**Published:** 2009-08-06

**Authors:** Altacílio A Nunes

**Affiliations:** Department of Social Medicine, University of Sao Paulo, Avenida Bandeirantes3900 - Monte Alegre, 14.048-900 - Ribeirao Preto SPBrazil

## Abstract

**Introduction:**

Toxoplasmosis, a zoonotic protozoal disease caused by *toxoplasma gondii*, is prevalent throughout the world, affecting a large proportion of persons who usually have no symptoms. Glucose 6 phosphate dehydrogenase deficiency, an X-linked inherited disorder, is present in over 400 million people world wide. It is more common in tropical and subtropical countries and is one of the important causes of hemolytic anemia.

**Case presentation:**

This case report relates the occurrence of the two diseases simultaneously in a child of five years old.

**Conclusion:**

Patients with glucose-6-phosphate dehydrogenase deficiency are more susceptible to toxoplasmosis and this case report, reinforce the findings of this propensity and alert us for such possibility, what it is important, therefore, the treatment of toxoplasmosis can cause serious hemolysis in these patients.

## Introduction

Toxoplasmosis is a disease generated by *toxoplasma gondii*, a protozoan parasite that infects up to a third of the world's population. Infection is mainly acquired by ingestion of food or water that is contaminated with oocysts shed by cats or by eating undercooked or raw meat containing tissue cysts. Primary infection is usually sub-clinical but in some patients, cervical lymphadenopathy or ocular disease can be present. In most adults and children it does not cause serious illness, but it can cause blindness and mental retardation in congenitally infected children and devastating disease in immunocompromised individuals [1,2]. There is report of precocious puberty as an endocrine manifestation in congenital toxoplasmosis [3].

Glucose-6-phosphate dehydrogenase (G-6-PD) deficiency, the most common enzyme deficiency worldwide, causes a spectrum of disease including neonatal hyperbilirubinemia, with acute and chronic hemolysis. Persons with this condition also may be asymptomatic. This X-linked inherited disorder most commonly affects persons of African, Asian, Mediterranean, or Middle-Eastern descent. Approximately 400 million people are affected worldwide [4,5].

Here is related a case of patient with acquired toxoplasmosis and glucose-6-phosphate dehydrogenase simultaneously.

## Case presentation

A 5 years old male child, white race, natural of Minas Gerais State, Brazil, presented with fatigue, malaise and low-grade fever at least 10 days. Examination revealed: pallor, painless axillary and cervical lymphadenopathy and splenohepatomegaly. His hemogram showed leucocytosis with anemia [Hb = 8.0 gm%, WBC = 28,200 cells/cumm (51% polymorphs and 49% lymphocytes)], his liver function tests were normal. At the thirteen day a clinical diagnosis of toxoplasmosis was considered, an enzyme-linked immunoassay (ELISA) for *toxoplasma* antigens in blood showed IgM = 1:256 and IgG = 1:1024. Ahead of the results the treatment with pyrimethamine 1 mg/kg/d plus sulfadiazine** (**100 mg/kg/day), plus folinic acid was initiated.

**Figure 1.**
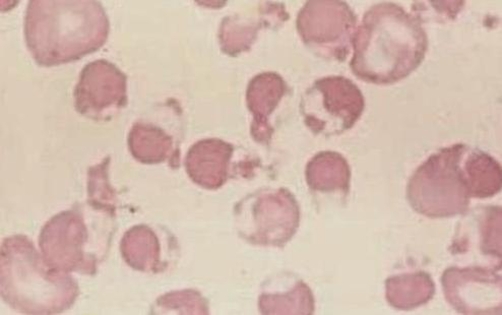
Hemolysis due to G-6-PD deficiency in a child of five years old.

In the seventh day of treatment the patient presented important jaundice and dark urine beyond decreased urine output, in the physical examination was observed, important splenohepatomegaly, tachycardia and tachypnea. A clinical diagnosis of acute hepatitis secondary to medications was considered. Laboratory exams showed: hemoglobin 6.4 gm%, WBC = 10,200 cells/cumm, serum bilirubin = 1.4 mg/dl with indirect bilirubin = 0.9 mg/dl, liver enzymes were normal, thus, a clinical diagnosis of acute hemolysis was considered and new examinations had shown reticulocyte count = 5.1% with corrected reticulocyte count = 1.7%, direct coombs test and indirect coombs test being negative, urine routine examination showed was normal with absent bile salts and bile pigments and urine culture did not show any organism. A smear of the blood showed an important degree of hemolysis (Figure 1). The patient was screened for G-6-PD activity, which was absent. Thus a diagnosis of acute acquired toxoplasmosis with G-6-PD deficiency was considered with acute hemolysis due to sulfadiazine ingestion.

The sulfadiazine was discontinued and the treatment was changed for clindamycin so the patient was discharged without jaundice with hemoglobin 10.0 gm% and reticulocyte count = 0.5%. On follow up after 1 month, his hemoglobin was 12.5 gm%.

The parents of patient were advised to avoid antimalarials, sulfa drugs, chloramphenicol, nitrofurantoin, nalidixic acid, Vitamin K and others drugs due to his G-6-PD deficiency state.

## Discussion

An unusual propensity for infection with catalase-positive organisms has been reported in patients with severe deficiency or complete absence of G-6-PD [4-6], however, propensity for infection with *toxoplasma gondii* was reported in only in a study [6] which showed an increased risk of *toxoplasma* infection by 2.5 folds in persons with G-6-PD deficiency as compared to G-6-PD normal individuals. The likely explanation for the increased infection rate is due to both direct destruction of the reticuloendothelial system by *toxoplasma* organisms and decreased killing effect of the phagocytic cells [6,7].

In this case report the presence of the two diseases in a child, reinforce the findings of this propensity and alert us for such possibility, mainly considering the high prevalence of the two illnesses in the world. World-wide prevalence of toxoplasmosis varies of 2.3% to 93%, while that the prevalence of G-6-PD deficiency varies of 0 to 27%, so, ahead of the necessity of treatment for toxoplasmosis this fact must be taken in consideration, for the possibility of hemolysis secondary to sulfas.

Clinical presentations of G-6-PD deficiency are secondary at some factors such as infections, intake of fava beans, antimicrobial drugs, mainly sulfas and its derivatives. In the case above reported, the patient was using sulfadiazine for treatment of toxoplasmosis and developed acute hemolysis, at which quickly decreased after removed of the medicine and its exchange for clindamycin, at which is usually recommended for treatment of toxoplasmosis in specials situations [6-8].

## Abbreviations

G-6-PD: Glucose-6-phosphate dehydrogenase
WBC: white blood cells
